# Supplementary material for: Paediatric cancer burden in Namibia: A 10-year retrospective, analytical cohort study of patients admitted at Windhoek Central Hospital
Source: PLoS One. 2023 Nov 16;18(11):e0292794. doi: 10.1371/journal.pone.0292794 (PMC10653541; doi:10.1371/journal.pone.0292794)
Supplement: S1 File — (DOCX) [file pone.0292794.s003.docx]

**Type of cancer diagnosed in comparison with diagnostic method**

|  | | | | | | | |
| --- | --- | --- | --- | --- | --- | --- | --- |
|  | | | **Diagnostic method** | | | | **Total** |
|  |  |  | **Clinical, Imaging & Histology** | **Histology** | **Imaging** | **Imaging & Histology** |  |
| **Type of cancer diagnosed** | **Brain or CNS tumor** | Count | 0 | 0 | 3 | 3 | 6 |
|  |  | % within Type of cancer diagnosed | 0.0% | 0.0% | 50.0% | 50.0% | 100.0% |
|  |  | % within Diagnostic method | 0.0% | 0.0% | 42.9% | 8.1% | 3.4% |
|  |  | % of Total | 0.0% | 0.0% | 1.7% | 1.7% | 3.4% |
|  | **Embryonal tumor** | Count | 3 | 32 | 3 | 27 | 65 |
|  |  | % within Type of cancer diagnosed | 4.6% | 49.2% | 4.6% | 41.5% | 100.0% |
|  |  | % within Diagnostic method | 75.0% | 25.4% | 42.9% | 73.0% | 37.4% |
|  |  | % of Total | 1.7% | 18.4% | 1.7% | 15.5% | 37.4% |
|  | **Haematopoetic tumors** | Count | 0 | 77 | 0 | 2 | 79 |
|  |  | % within Type of cancer diagnosed | 0.0% | 97.5% | 0.0% | 2.5% | 100.0% |
|  |  | % within Diagnostic method | 0.0% | 61.1% | 0.0% | 5.4% | 45.4% |
|  |  | % of Total | 0.0% | 44.3% | 0.0% | 1.1% | 45.4% |
|  | **Soft tissue and bone tumor** | Count | 1 | 17 | 1 | 5 | 24 |
|  |  | % within Type of cancer diagnosed | 4.2% | 70.8% | 4.2% | 20.8% | 100.0% |
|  |  | % within Diagnostic method | 25.0% | 13.5% | 14.3% | 13.5% | 13.8% |
|  |  | % of Total | 0.6% | 9.8% | 0.6% | 2.9% | 13.8% |
| **Total** | | Count | 4 | 126 | 7 | 37 | 174 |
|  |  | % within Type of cancer diagnosed | 2.3% | 72.4% | 4.0% | 21.3% | 100.0% |
|  |  | % within Diagnostic method | 100.0% | 100.0% | 100.0% | 100.0% | 100.0% |
|  |  | % of Total | 2.3% | 72.4% | 4.0% | 21.3% | 100.0% |
