# Supplementary figures and images for: Paediatric cancer burden in Namibia: A 10-year retrospective, analytical cohort study of patients admitted at Windhoek Central Hospital
Source: PLoS One. 2023 Nov 16;18(11):e0292794. doi: 10.1371/journal.pone.0292794 (PMC10653541; doi:10.1371/journal.pone.0292794)

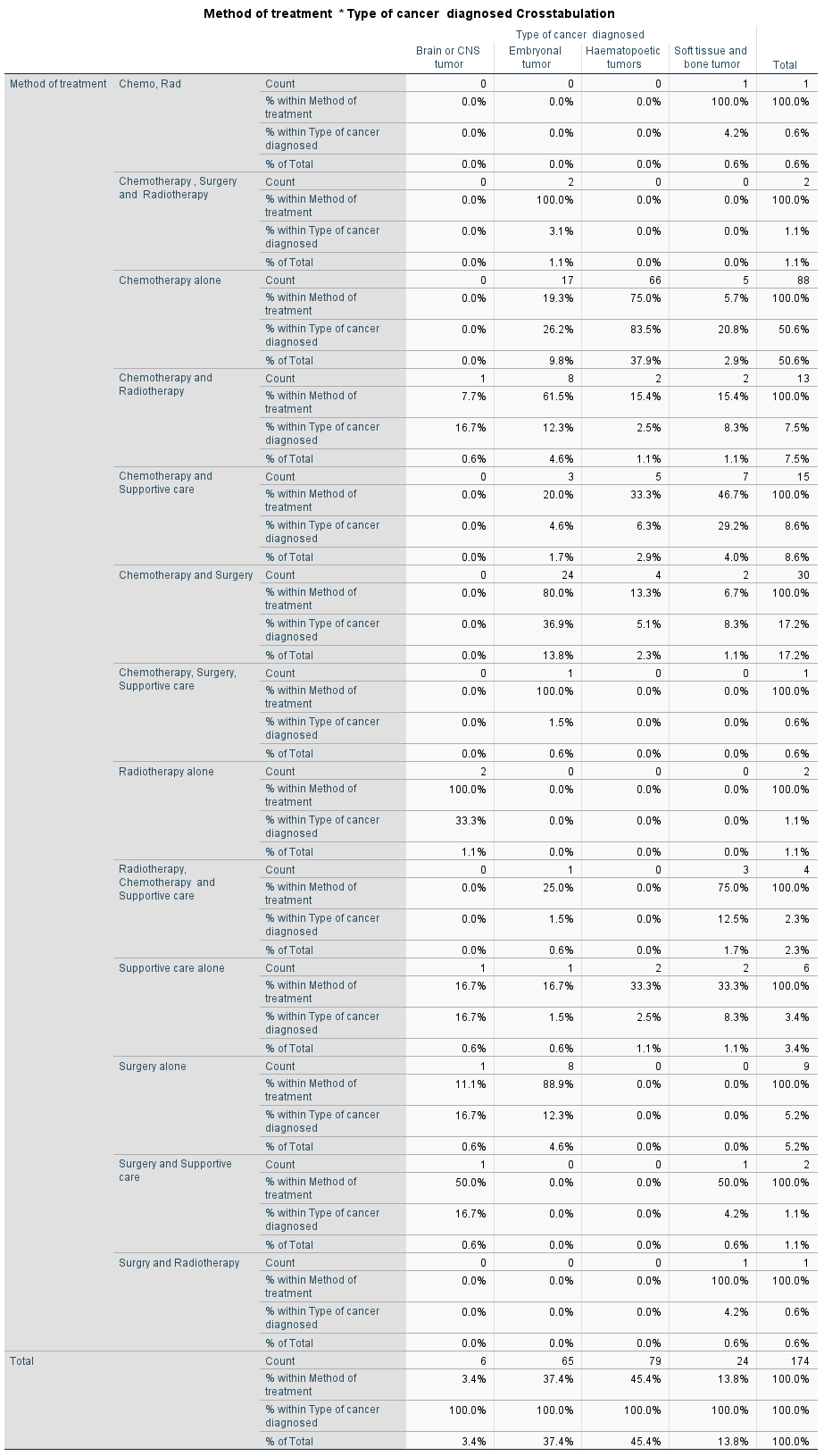

Supplement: S2 File — (DOCX) [file pone.0292794.s004.docx]
