# Supplementary material for: Paediatric cancer burden in Namibia: A 10-year retrospective, analytical cohort study of patients admitted at Windhoek Central Hospital
Source: PLoS One. 2023 Nov 16;18(11):e0292794. doi: 10.1371/journal.pone.0292794 (PMC10653541; doi:10.1371/journal.pone.0292794)
Supplement: S3 File — Length of hospital stay and cancer type frequencies and percentages for paediatric participants admitted at an oncology ward. (DOCX) [file pone.0292794.s005.docx]

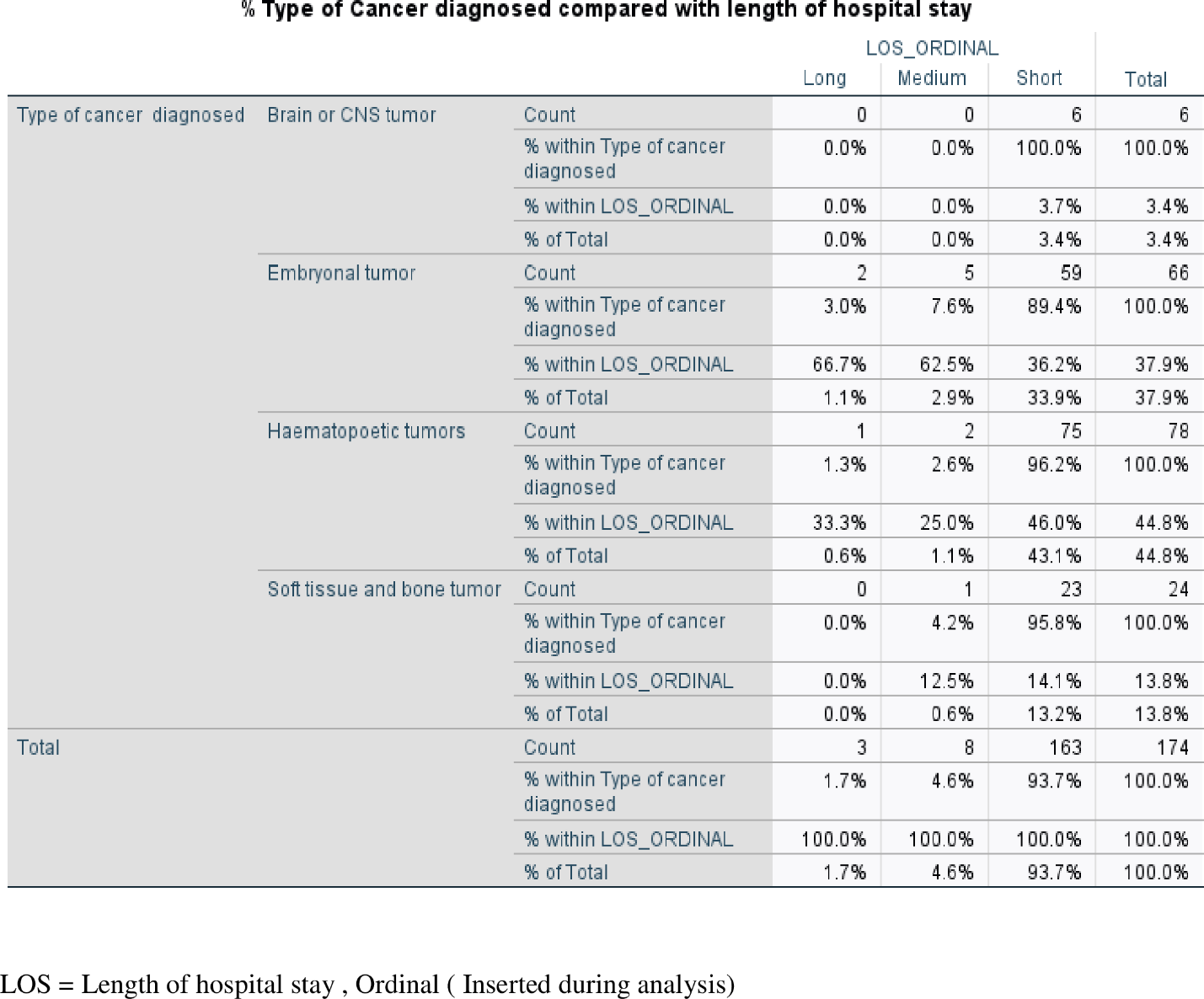


**Supplementary 3**: Length of hospital stay and cancer type frequencies and percentages for paediatric participants admitted at an oncology ward.

LOS = Length of hospital stay , Ordinal ( Inserted during analysis)
