# Supplementary material for: Paediatric cancer burden in Namibia: A 10-year retrospective, analytical cohort study of patients admitted at Windhoek Central Hospital
Source: PLoS One. 2023 Nov 16;18(11):e0292794. doi: 10.1371/journal.pone.0292794 (PMC10653541; doi:10.1371/journal.pone.0292794)
Supplement: S4 File — (PDF) [file pone.0292794.s006.pdf]

REPUBLIC OF NAMIBIA

Ministry of Health and Social Services

Private Bag 13215
Windhoek
Namibia
Enquiries: Mrs. S. Iipinge

Harvey Street
Windhoek
Ref. RNM 2021

Tel. No: (061) 203 3024
Fax No: (061) 222006
Date: 06 July 2021

**OFFICE OF THE CHIEF MEDICAL SUPERINTENDENT
WINDHOEK CENTRAL HOSPITAL**

Ms. Ndapewa O. Kaholongo
School Of Medicine
University Of Namibia
0813100803

SUBJECT: PERMISSION TO CONDUCT THE RESEARCH STUDY ON THE PATTERN OF PAEDIATRIC CANCER PATIENTS ADMITTED AT WINDHOEK CENTRAL HOSPITAL:A 10 YEAR REVIEW

1. Reference is made to your application to conduct the above-mentioned study.

2. This letter serves to inform you that permission has been granted for you to conduct a study at Windhoek Central Hospital, on the above mentioned subject as you have requested and does not include any remuneration.

3. Patient/Client's information should be kept confidential at all times.

4. Preliminary findings to be submitted to Customer care office, Windhoek Central Hospital upon completion of the study.

Thank you for your kind gesture.

Yours sincerely

....

DR. D.I.UIRAB
CHIEF MEDICAL SUPERINTENDENT

MINISTRY OF HEALTH AND SOCIAL SERVICES
Private Bag 13216
08 -07- 2021
[illegible] Namibia
[illegible] HOSPITAL

"*Your Health, Our Concern*"
