# Supplementary material for: Paediatric cancer burden in Namibia: A 10-year retrospective, analytical cohort study of patients admitted at Windhoek Central Hospital
Source: PLoS One. 2023 Nov 16;18(11):e0292794. doi: 10.1371/journal.pone.0292794 (PMC10653541; doi:10.1371/journal.pone.0292794)
Supplement: S5 File — (PDF) [file pone.0292794.s007.pdf]

REPUBLIC OF NAMIBIA

MINISTRY OF HEALTH AND SOCIAL SERVICES

OFFICE OF THE EXECUTIVE DIRECTOR

Ministerial Building
Harvey Street
Private Bag 13198, Windhoek

Tel: No: 061 -203 2507
Fax No: 061-222 558
Andreas.Shipanga@mhss.gov.na

Ref: 22/4/2/3
Enquiries: Mr. A. Shipanga

Date: 03 October 2022

Ms. Ndapewa Kaholongo
PO Box 50451
Bachbrecht
Windhoek

Dear Ms. Kaholongo

Re: Approval for permission to publish a case report title "Paediatric cancer burden in Namibia, a 10 year retrospective, analytical study of patients admitted at Windhoek Central Hospital, Namibia".

1. The above matter has reference.

2. Kindly be informed that approval *for* permission to publish a case report *"Paediatric cancer burden in Namibia, a 10 year retrospective, analytical study of patients admitted at Windhoek Central Hospital, Namibia"* has been granted.

3. **The study mentioned above should only be published in the following Journals:**
   - BioMed Central (BMC) Pediatrics,
   - The Public Library of Science (PLOS) Journal
   - The Undergraduate Research in Health Journal (URHJ)
   - Journal of Public Health in Africa (JHIA).

Yours sincerely,

EXECUTIVE DIRECTOR
MINISTRY OF HEALTH AND SOCIAL SERVICES

BEN NANGOMBE
EXECUTIVE DIRECTOR
