# Supplementary material for: Paediatric cancer burden in Namibia: A 10-year retrospective, analytical cohort study of patients admitted at Windhoek Central Hospital
Source: PLoS One. 2023 Nov 16;18(11):e0292794. doi: 10.1371/journal.pone.0292794 (PMC10653541; doi:10.1371/journal.pone.0292794)
Supplement: Attachment — Submitted filename: Response to the reviewers Final.docx [file pone.0292794.s008.docx]

Response to the reviewers

To the Editor

Firstly, we would like to thank The PLOS ONE for considering our manuscript "PONE-D-23-09242: Paediatric cancer burden in Namibia: A 10-year retrospective, analytical cohort study of patients admitted at Windhoek Central Hospital” for review. Secondly, we appreciate the reviewers’ and Editor’s comments. The authors responded to comments and the authors’ changes are highlighted in the main manuscript labelled “Revised manuscript with track changes”. Below are the responses to the reviewers in a table form.

| **Journal Requirements Concerns Raised** | **Authors’ Responses** |
| --- | --- |
| 1. Please ensure that your manuscript meets PLOS ONE's style requirements, including those for file naming. The PLOS ONE style templates can be found at  <https://journals.plos.org/plosone/s/file?id=wjVg/PLOSOne_formatting_sample_main_body.pdf> and  <https://journals.plos.org/plosone/s/file?id=ba62/PLOSOne_formatting_sample_title_authors_affiliations.pdf> | Thank you. The authors have attended to this. |
| 2. 2. We suggest you thoroughly copyedit your manuscript for language usage, spelling, and grammar. If you do not know anyone who can help you do this, you may wish to consider employing a professional scientific editing service.  Whilst you may use any professional scientific editing service of your choice, PLOS has partnered with both American Journal Experts (AJE) and Editage to provide discounted services to PLOS authors. Both organizations have experience helping authors meet PLOS guidelines and can provide language editing, translation, manuscript formatting, and figure formatting to ensure your manuscript meets our submission guidelines. To take advantage of our partnership with AJE, visit the AJE website (<http://learn.aje.com/plos/>) for a 15% discount off AJE services. To take advantage of our partnership with Editage, visit the Editage website ([www.editage.com](http://www.editage.com)) and enter referral code PLOSEDIT for a 15% discount off Editage services.  If the PLOS editorial team finds any language issues in text that either AJE or Editage has edited, the service provider will re-edit the text for free.  Upon resubmission, please provide the following:   The name of the colleague or the details of the professional service that edited your manuscript  A copy of your manuscript showing your changes by either highlighting them or using track changes (uploaded as a *supporting information* file)  A clean copy of the edited manuscript (uploaded as the new *manuscript* file)” | AJE was used to copyedit the manuscript. Secondly we approached Dr  Tonny. P. Tauro (Doctoral Fellow, M.Phil, BSc)  Lecturer (Soil Science) Marondera University of Agricultural Sciences and Technology  P.O Box 35, Marondera,  Zimbabwe  Email: phirilani2@yahoo.co.uk |
| 3. We note that you have stated that you will provide repository information for your data at acceptance. Should your manuscript be accepted for publication, we will hold it until you provide the relevant accession numbers or DOIs necessary to access your data. If you wish to make changes to your Data Availability statement, please describe these changes in your cover letter and we will update your Data Availability statement to reflect the information you provide. | Thank you for highlighting the need for us to provide the DOI for the data sets. The data sets can be accessed through ZENODO on the following link: <https://zenodo.org/badge/DOI/10.5281/zenodo.8270769.svg>.  Mentioned under availability of data lines 474-475 |
| 4. Your ethics statement should only appear in the Methods section of your manuscript. If your ethics statement is written in any section besides the Methods, please delete it from any other section. | Thank you for the comments. The ethics statement was removed from the declaration section and now only appears in the Methods section. |
| 5. We note that Figure 1 in your submission contain map images which may be copyrighted. All PLOS content is published under the Creative Commons Attribution License (CC BY 4.0), which means that the manuscript, images, and Supporting Information files will be freely available online, and any third party is permitted to access, download, copy, distribute, and use these materials in any way, even commercially, with proper attribution. For these reasons, we cannot publish previously copyrighted maps or satellite images created using proprietary data, such as Google software (Google Maps, Street View, and Earth). For more information, see our copyright guidelines: <http://journals.plos.org/plosone/s/licenses-and-copyright>..   We require you to either (1) present written permission from the copyright holder to publish these figures specifically under the CC BY 4.0 license, or (2) remove the figures from your submission:   a. You may seek permission from the original copyright holder of Figure 1 to publish the content specifically under the CC BY 4.0 license.     We recommend that you contact the original copyright holder with the Content Permission Form (<http://journals.plos.org/plosone/s/file?id=7c09/content-permission-form.pdf>) and the following text: “I request permission for the open-access journal PLOS ONE to publish XXX under the Creative Commons Attribution License (CCAL) CC BY 4.0 (<http://creativecommons.org/licenses/by/4.0/>). Please be aware that this license allows unrestricted use and distribution, even commercially, by third parties. Please reply and provide explicit written permission to publish XXX under a CC BY license and complete the attached form.”   Please upload the completed Content Permission Form or other proof of granted permissions as an "Other" file with your submission.   In the figure caption of the copyrighted figure, please include the following text: “Reprinted from [ref] under a CC BY license, with permission from [name of publisher], original copyright [original copyright year].”   b. If you are unable to obtain permission from the original copyright holder to publish these figures under the CC BY 4.0 license or if the copyright holder’s requirements are incompatible with the CC BY 4.0 license, please either i) remove the figure or ii) supply a replacement figure that complies with the CC BY 4.0 license. Please check copyright information on all replacement figures and update the figure caption with source information. If applicable, please specify in the figure caption text when a figure is similar but not identical to the original image and is therefore for illustrative purposes only. The following resources for replacing copyrighted map figures may be helpful:   USGS National Map Viewer (public domain): <http://viewer.nationalmap.gov/viewer/> The Gateway to Astronaut Photography of Earth (public domain): <http://eol.jsc.nasa.gov/sseop/clickmap/> Maps at the CIA (public domain): <https://www.cia.gov/library/publications/the-world-factbook/index.html> and <https://www.cia.gov/library/publications/cia-maps-publications/index.html> NASA Earth Observatory (public domain): <http://earthobservatory.nasa.gov/> Landsat: <http://landsat.visibleearth.nasa.gov/> USGS EROS (Earth Resources Observatory and Science (EROS) Center) (public domain): [http://eros.usgs.gov/#](http://eros.usgs.gov/) Natural Earth (public domain): <http://www.naturalearthdata.com/>  [Note: HTML markup is below. Please do not edit.] | Thank you for the concern. We have replaced the map using the one downloaded from Maps at the CIA (public domain): <https://www.cia.gov/library/publications/the-world-factbook/index.html> and <https://www.cia.gov/library/publications/cia-maps-publications/index.html>  The reference for the new map is (13). |
|  |  |
| **REVIEWERS’ COMMENTS** | **AUTHORS’ RESPONSES** |
| 1. Is the manuscript technically sound, and do the data support the conclusions?  The manuscript must describe a technically sound piece of scientific research with data that supports the conclusions. Experiments must have been conducted rigorously, with appropriate controls, replication, and sample sizes. The conclusions must be drawn appropriately based on the data presented.  Reviewer #1: Partly | Thank you for the comments. The authors have revised their conclusion as well analyzing the data and this has been highlighted in the revised submission. |
| 2. Has the statistical analysis been performed appropriately and rigorously?  Reviewer #1: No | Thank you for the concerns. The authors noted the concerns and saw their gaps and the need to reanalyze the data. The data was reanalyzed including a Detrended correspondence analysis (DCA) diagrams of cancer type and environmental variable. New figures and tables were added: Figures 5 -8, Tables 6 & 7. |
| 3. Have the authors made all data underlying the findings in their manuscript fully available?  The [PLOS Data policy](http://www.plosone.org/static/policies.action#sharing) requires authors to make all data underlying the findings described in their manuscript fully available without restriction, with rare exception (please refer to the Data Availability Statement in the manuscript PDF file). The data should be provided as part of the manuscript or its supporting information, or deposited to a public repository. For example, in addition to summary statistics, the data points behind means, medians and variance measures should be available. If there are restrictions on publicly sharing data—e.g. participant privacy or use of data from a third party—those must be specified.  Reviewer #1: Yes | The authors had indicated that all the data for manuscript was included and that if there was need for further question with regards to data then the corresponding author was ready to answer any questions. However to improve visibility of the data, the dataset are now published in ZENODO and can be accessed via the following link: <https://zenodo.org/badge/DOI/10.5281/zenodo.8270769.svg>.  The availability of data section now reads as follows: ‘The datasets used and/or analysed during the current study are available from Zenodo and can be accessed via <https://zenodo.org/badge/DOI/10.5281/zenodo.8270769.svg>. ‘  Lines: 478-479 |
| 4. Is the manuscript presented in an intelligible fashion and written in standard English?  PLOS ONE does not copyedit accepted manuscripts, so the language in submitted articles must be clear, correct, and unambiguous. Any typographical or grammatical errors should be corrected at revision, so please note any specific errors here.  Reviewer #1: No | Thank you for the comments. The authors uploaded the manuscript for copy editing to AJE. In addition Mr Tonny Tauro was engaged to help with copyediting the manuscript. We hope the manuscript reads better now. |
|  |  |
| **REVIEWER #1 COMMENTS TO AUTHOR** | **AUTHORS’ RESPONSES** |
| **Abstract**: − The abstract has all the sections required for an abstract. The background should be revised, this study is focused on paediatric cancer burden and not on the scarcity of research in paediatric oncology. The background focus should be on paediatric cancer burden | Thank you for the input. We have corrected this point to say that there is a constant need to have updated statistics on the changing trends in the frequency of different types of cancers to inform policy hence the reason for the current study. The abstract was reworded and following the analysis of data new information was added to the abstract. Line 48-77 |
| − **Method** is not cross-sectional analysis. | Thank you. The authors have gladly revised the method. It is now an analytical retrospective cohort study. |
| **Background:** − Background need to be revised, the focus is on paediatric cancer, but there is repetition of ideas, the last paragraph should be included in the first part of this abstract. The second paragraph is providing justification for carrying out this study, therefore it should be the last and paragraph in the background which should also indicate the objective of carrying out this study. − There are redundant statements which need to be omitted or revised to show their relevance. A good example of this is this statement ‘Namibia is a diverse nation of approximately 2.59 million people’  − The order of references in this section is awkward, the references need to be arranged in ascending sequence. | Thank for the concern. The authors that the references needed revision and follow the proper Vancouver style and that some statements were redundant. As a follow up to this the background section was revised and proper referencing performed. Lines: 95-142.  ‘Namibia is a diverse nation of approxi mately 2.59 million people’: now reads  ‘’Namibia a diverse nation of approximately 2.59 million people had an estimated number 11 248 Namibians diagnosed with a malignant neoplasms in the last Namibian National Cancer Registry in 2014’’ Lines 108-110  The references have now been arranged with an ascending order. Thank you for the comments. |
| **Method:** This section need to be revised and more information are required, the following information need to be clarified − When was data collected, exact months and year for this activity?  − There are should be clarification of the ethical approval for this study. o Which Review board provided ethical approval for this study? o  This was a retrospective study, why did the MOHSS and matron give consent for this study?  Why is it stated that there was a waiver of consent which was given.    The setting for this study should be briefly described, who is providing care for this children with oncology, how big is the unit, how many children are admitted at one time. These information are useful to give context to the reader of this work. | Thank you. The authors have revised this section.  Line 164 now reads: ‘The research data was collected between July 2021 and September 2022’.    The public hospitals in Namibia are under the management of the Ministry of Health and Social Services. Within this Ministry there is a department for research responsible for providing ethical approval to any research involving human subjects. Ethical approval for the study therefore was obtained from the Ministry of Health and Social Services (MOHSS)’s Biomedical Research Ethics Committee (BREC) and Research Management Committee (RMC) in the Office of the Executive Director (reference number NOK 2021). Of note, MOHSS’s Biomedical Research Ethics Committee (BREC) is an entity under MOHSS.  The matron of the hospital did not provide consent but rather gave permission for the researcher to be in the ward as she is the ward manager.  The waiver was provided to the study because it involved use of records rather than real patients as per the ministry of health policy.  The setting of the study was briefly described now in the revised manuscript. Line 156-161 “The hospital is situated in the capital city of Namibia and caters for patients from all regions as far as more than 800 kilometers away. Additionally, the hospital has a total bed capacity of 855 patients including oncology patients (the total bed capacity of the oncology ward 8 West is 29). The paediatric oncology is manned by general paediatricians and medical officers and has no resident oncologist.’ |
| **Results** The data presented shows a clear spectrum of paediatric cancers in Namibia. The following should be added or reorganized for clarity and improvement of results  − Data on histology should be included in the results especially for solid organ cancers.    − Method of diagnosing cancers should be based on categories of cancers. It is very difficult to understand and make sense out of the results. It will be interesting to know how the diagnosis was made for brain tumours, haematologic cancers etc. − Treatment should be categorized for each group of tumours  − Hospital stay may also be provided for each group of cancers as well eg. Haematologic cancers.    When reporting data use only a single decimal point.  Is there any relevance of showing regions from which patients came from in this report? | Thank you for the concerns raised. The authors have made the necessary changes.  Data was reanalyzed and new figures and tables where necessary made.  The actual histology findings were not reported but the information was whether or not histology was done for diagnostic purposes  A detailed figure Supplementary file 2 is attached on type of cancer diagnosed and diagnostic method. Fig 4. Line 288.  Treatment for each tumour was categorized. Lines 314-334. Fig 6 illustrates the type of cancer diagnosed and the treatment method used.  For more information a detailed supplementary file 3 is attached.  A section on length of hospital stay was modified and additional Table 7 and Fig 7 added. Further detailed Supplementary file 3 is attached for addition information.  Lines 338-351  One decimal point was used for the data instead of the two used previously  The region of origin are relevant to the study to indicate how far the patients are coming from. Additionally, this can inform policy to decentralize treatment to the regions with highest number of patients. Some patients have to travel as far as 800km to access the central hospital. |
| **Discussion** In the discussion the authors are using their data to conclude on differences between ages and sex with occurrence of cancers. This is not possible as in the analysis and results no statistical analysis was carried out. This is a descriptive study for all variables related to cancer, and no analysis was carried out and therefore no difference was observed. | Thank you for the input. The authors have reanalyzed some of the data and worked on the discussion to make a comparison rather than just describing. |
| While revising your submission, please upload your figure files to the Preflight Analysis and Conversion Engine (PACE) digital diagnostic tool, <https://pacev2.apexcovantage.com/>. PACE helps ensure that figures meet PLOS requirements. To use PACE, you must first register as a user. Registration is free. Then, login and navigate to the UPLOAD tab, where you will find detailed instructions on how to use the tool. If you encounter any issues or have any questions when using PACE, please email PLOS at [figures@plos.org](mailto:figures@plos.org). Please note that Supporting Information files do not need this step. | The figure files were uploaded to the Preflight analysis and Conversion Engine (PACE) and the converted images were then downloaded to be placed back in the manuscript. |
